# A Dual Formula for the Noncommutative Transport Distance

**DOI:** 10.1007/s10955-022-02911-9

**Published:** 2022-04-08

**Authors:** Melchior Wirth

**Affiliations:** Institute of Science and Technology Austria (ISTA), Am Campus 1, 3400 Klosterneuburg, Austria

**Keywords:** Quantum Markov semigroup, Duality, Quantum optimal transport

## Abstract

In this article we study the noncommutative transport distance introduced by Carlen and Maas and its entropic regularization defined by Becker and Li. We prove a duality formula that can be understood as a quantum version of the dual Benamou–Brenier formulation of the Wasserstein distance in terms of subsolutions of a Hamilton–Jacobi–Bellmann equation.

## Introduction

The theory of optimal transport [28, 29] has experienced rapid growth in recent years with applications in diverse fields across pure and applied mathematics. Along with this growth came a lot of interest in extending the methods of optimal transport beyond the scope of its original formulation as an optimization problem for the transport cost between two probability measures.

One such extension deals with “quantum spaces”, where the probability measures are replaced by density matrices or density operators. Most of the work on quantum optimal transport in this sense can be grouped into one of the following two categories. The first approach (see e.g. [6–9, 11, 22, 27]) takes a quantum Markov semigroup (QMS) as input datum and relies on a noncommutative analog of the Benamou–Brenier formulation [4] of the Wasserstein distance for probability measures on Euclidean space

$$\begin{aligned} W_2^2(\mu ,\nu )=\inf \left\{ \int _0^1\int _{\mathbb {R}^n}|v_t|^2\,d\rho _t\,dt: \rho _0=\mu ,\rho _1=\nu ,\dot{\rho }_t+\nabla \cdot (\rho _t v_t)=0\right\} . \end{aligned}$$

In the simple case when the generator $\mathscr {L}$ of the QMS is of the form

$$\begin{aligned} \mathscr {L}A=\sum _{j\in \mathcal {J}}[V_j,[V_j,A]] \end{aligned}$$

with self-adjoint matrices $V_j$, the associated noncommutative transport distance $\mathcal {W}$ on the set of density matrices is given by

$$\begin{aligned} \mathcal {W}^2(\rho _0,\rho _1)=\inf \left\{ \int _0^1 \sum _{j\in \mathcal {J}}\tau (\mathbf {W}_j^*(t) [\rho (t)]_0^{-1}(\mathbf {W}_j))\,dt: \dot{\rho }(t)=\sum _{j\in \mathcal {J}}[V_j,\mathbf {W}_j(t)]\right\} , \end{aligned}$$

where the infimum is taken over curves $\rho$ that satisfy $\rho (0)=\rho _0$, $\rho (1)=\rho _1$, and where

$$\begin{aligned} {[}X]_0(A)=\int _0^1 X^\alpha A X^{1-\alpha }\,d\alpha . \end{aligned}$$

For the definition of the metric $\mathcal {W}$ in the more general case of a QMS satisfying the detailed balance condition (DBC), we refer to the next section.

This approach has proven fruitful in applications to noncommutative functional inequalities, similar in spirit to the heuristics known as Otto calculus [8, 9, 12, 31].

The second approach (see e.g. [13, 14, 17, 23, 25, 26]) seeks to find a suitable noncommutative analog of the Monge–Kantorovich formulation [20] of the Wasserstein distance via couplings (or transport plans):

$$\begin{aligned} W_p^p(\mu ,\nu )=\inf \left\{ \int _{X\times X}d^p(x,y)\,d\pi (x,y):(\mathrm {pr}_1)_\#\pi =\mu ,(\mathrm {pr}_2)_\#\pi =\nu \right\} . \end{aligned}$$

This approach also allows to consider a quantum version of Monge–Kantorovich problem for arbitrary cost functions. So far, possible connections between these two approaches in the quantum world stay elusive.

The focus of this article lies on the noncommutative transport distance $\mathcal {W}$ introduced in the first approach. More precisely, we prove a dual formula that is a noncommutative analog of the expression of the classical $L^2$-Wasserstein distance in terms of subsolutions of the Hamilton–Jacobi equation [5, 24]

$$\begin{aligned} W_2^2(\mu ,\nu )=\frac{1}{2} \inf \left\{ \int _{\mathbb {R}^n} u_1\,d\mu -\int _{\mathbb {R}^n}u_0\,d\nu : \dot{u}_t+\frac{1}{2}|\nabla u_t|^2\le 0\right\} . \end{aligned}$$

This result yields a noncommutative version of the dual formula obtained independently by Erbar et al. [15] and Gangb et al. [16] for the Wasserstein-like transport distance on graphs. In fact, we prove a dual formula that is not only valid for the metric $\mathcal {W}$, but also for the entropic regularization recently introduced by Becker–Li [3]. When the generator $\mathscr {L}$ is again of the simple form discussed above, the entropic regularization $\mathcal {W}_\varepsilon$ is a metric obtained when replacing the constraint

$$\begin{aligned} \dot{\rho }(t)=\sum _{j\in \mathcal {J}}[V_j,\mathbf {W}_j(t)] \end{aligned}$$

in the definition of $\mathcal {W}$ by

$$\begin{aligned} \dot{\rho }(t)=\sum _{j\in \mathcal {J}}[V_j,\mathbf {W}_j(t)]+\varepsilon \mathscr {L}^\dagger \rho (t). \end{aligned}$$

With the notation introduced in the next section, the main result of this article reads as follows.

### Theorem

Let $\sigma \in M_n(\mathbb {C})$ be an invertible density matrix and $(P_t)$ an ergodic QMS on $M_n(\mathbb {C})$ that satisfies the $\sigma$-DBC. The entropic regularization $\mathcal {W}_\varepsilon$ of noncommutative transport distance induced by $(P_t)$ satisfies the following dual formula:

$$\begin{aligned} \frac{1}{2}\mathcal {W}_\varepsilon ^2(\rho _0,\rho _1)=\sup \{\tau (A(1)\rho _1-A(0)\rho _0)\mid A\in \mathsf {HJB}^1_{\varepsilon }\}. \end{aligned}$$

Here a QMS $(P_t)$ is said to satisfy the $\sigma$-DBC if

$$\begin{aligned} \tau ((P_t A)^*B\sigma )=\tau (A^*(P_t B)\sigma ) \end{aligned}$$

for all $A,B\in M_n(\mathbb {C})$ and $t\ge 0$. If $\sigma$ is the identity matrix, this is the case exactly when the generator is of the form $\mathscr {L}A=\sum _j [V_j,[V_j,A]]$ with self-adjoint matrices $V_j$.

Moreover, $\mathsf {HJB}^1_{\varepsilon }$ stands for the set of all Hamilton–Jacobi–Bellmann subsolutions, a suitable noncommutative variant of solutions of the differential inequality

$$\begin{aligned} \dot{u}(t)+\frac{1}{2}|\nabla u(t)|^2-\varepsilon \Delta u(t)\le 0. \end{aligned}$$

Other metrics similar to $\mathcal {W}$ also occur in the literature, most notably the one called the “anticommutator case” in [3, 10, 11]. In [9, 30], a class of such metrics was studied in a systematic way, and our main theorem applies in fact to this wider class of metrics. For the anticommutator case, this duality formula was obtained before in [10].

There are still some very natural questions left open. For one, we do not discuss the existence of optimizers. While for the primal problem this follows from a standard compactness argument, this question is more delicate for the dual problem, even when dealing with probability densities on discrete spaces instead of density matrices, and one has to relax the problem to obtain maximizers (see [16,  Sects. 6–7]).

Another interesting direction would be to extend the duality result from matrix algebras to infinite-dimensional systems. While a definition of the metric $\mathcal {W}$ for QMSs on semi-finite von Neumann algebras is available [19, 30], the problem of duality seems to be much harder to address. Even for abstract diffusion semigroups, the best known result only shows that the primal distance is the upper length distance associated with the dual distance and leaves the question of equality open [2,  Proposition 10.11].

## Setting and Basic Definitions

In this section we introduce basic facts and definitions about QMSs that will be used later on. In particular, we review the definition of the noncommutative transport distance from [8] and its entropic regularization introduced in [3]. Our notation mostly follows [8, 9]. For a list of symbols we refer the reader to the end of this article.

Let $M_n(\mathbb {C})$ denote the complex $n\times n$ matrices and let $\mathcal {A}$ be a unital $*$-subalgebra of $M_n(\mathbb {C})$. Let $\mathcal {A}_h$ denote the self-adjoint part of $\mathcal {A}$, $\mathcal {A}_+$ the cone of positive elements of $\mathcal {A}$ and $\mathcal {A}_{++}$ the subset of invertible positive elements. We write $\tau$ for the normalized trace on $M_n(\mathbb {C})$, that is,

$$\begin{aligned} \tau (A)=\frac{1}{n}\sum _{k=1}^n A_{kk}, \end{aligned}$$

and $\mathfrak {H}_\mathcal {A}$ for the Hilbert space formed by equipping $\mathcal {A}$ with the GNS inner product

$$\begin{aligned} \langle \cdot ,\cdot \rangle _{\mathfrak {H}_A}:\mathcal {A}\times \mathcal {A}\rightarrow \mathbb {C},\quad (A,B)\mapsto \tau (A^*B). \end{aligned}$$

The adjoint of a linear operator $\mathscr {K}:\mathfrak {H}_\mathcal {A}\rightarrow \mathfrak {H}_\mathcal {A}$ is denoted by $\mathscr {K}^\dagger$.

We write $\mathfrak {S}(\mathcal {A})$ for the set of all density matrices on $\mathcal {A}$, that is, all positive elements $\rho \in \mathcal {A}$ with $\tau (\rho )=1$. The subset of invertible density matrices is denoted by $\mathfrak {S}_+(\mathcal {A})$.

A QMS on $\mathcal {A}$ is a family $(P_t)_{t\ge 0}$ of linear operators on $\mathcal {A}$ that satisfy the following conditions:

- $P_t$ is unital and completely positive for every $t\ge 0$,
- $$P_0=\mathrm {id}_\mathcal {A}$$, $$P_{s+t}=P_s P_t$$ for all $$s,t\ge 0$$,
- $$t\mapsto P_t$$ is continuous.

We consider a QMS $(P_t)$ on $\mathcal {A}$ which extends to a QMS on $M_n(\mathbb {C})$ satisfying the $\sigma$-detailed balance condition ($\sigma$-DBC) for some density matrix $\sigma \in \mathfrak {S}_+(\mathcal {A})$, that is,

$$\begin{aligned} \tau ((P_t A)^*B \sigma )=\tau (A^*(P_t B)\sigma ) \end{aligned}$$

for $A,B\in \mathcal {A}$ and $t\ge 0$. For $\sigma =\mathrm {id}_\mathcal {A}$, this reduces to the symmetry condition $P_t^\dagger =P_t$.

Let $\mathscr {L}$ denote the generator of $(P_t)$, that is, the linear operator on $\mathcal {A}$ given by

$$\begin{aligned} \mathscr {L}(A)=\lim _{t\searrow 0}\frac{P_t A-A}{t}. \end{aligned}$$

We further assume that $(P_t)$ is *ergodic* (or primitive), that is, the kernel of $\mathscr {L}$ is one-dimensional. This assumption is natural in this context as it ensures that the metric $\mathcal {W}_{\varvec{\Lambda ,\varepsilon }}$ defined below is the geodesic distance induced by a Riemannian metric on $\mathfrak {S}_+(\mathcal {A})$ and in particular that it is finite.

Generators of QMSs are often described by their Lindblad form, but here we will rely on the additional structure coming from the $\sigma$-DBC and use a presentation of $\mathscr {L}$ provided by Alicki’s theorem [1, Theorem 3], [8, Theorem 3.1] instead: There exists a finite set $\mathcal {J}$, real numbers $\omega _j$ for $j\in \mathcal {J}$ and $V_j\in M_n(\mathbb {C})$ for $j\in \mathcal {J}$ with the following properties:

- $$\tau (V_j^*V_k)=\delta _{jk}$$ for $$j,k\in \mathcal {J}$$,
- $$\tau (V_j)=0$$ for $$j\in \mathcal {J}$$,
- for every $j\in \mathcal {J}$ there exists a unique $j^*\in \mathcal {J}$ with $V_{j^*}=V_j^*$,
- $$\sigma V_j \sigma ^{-1}=e^{-\omega _j}V_j$$ for $$j\in \mathcal {J}$$

such that

$$\begin{aligned} \mathscr {L}(A)=\sum _{j\in \mathcal {J}}\left( e^{-\omega _j/2}V_j^*[A,V_j]-e^{\omega _j/2}[A,V_j]V_j^*\right) \end{aligned}$$

for $$A\in \mathcal {A}$$.

The numbers $\omega _j$ are called Bohr frequencies of $\mathscr {L}$ and are uniquely determined by $(P_t)$. The matrices $V_j$ are not uniquely determined by $(P_t)$ and $\sigma$, but in the following we will fix a set $\{V_j\mid j\in \mathcal {J}\}$ that satisfies the preceding conditions.

Next we will discuss how the data from Alicki’s theorem give rise to a differential structure associated with $\mathscr {L}$.

Let

$$\begin{aligned} \mathfrak {H}_{\mathcal {A},\mathcal {J}}=\bigoplus _{j\in \mathcal {J}}\mathfrak {H}_\mathcal {A}^{(j)}, \end{aligned}$$

where $\mathfrak {H}_\mathcal {A}^{(j)}$ is a copy of $\mathfrak {H}_\mathcal {A}$ for $j\in \mathcal {J}$. This is the quantum analog of the space of tangent vector fields in our setting.

We write $\partial _j$ for $[V_j,\cdot \,]$ and

$$\begin{aligned} \nabla :\mathfrak {H}_\mathcal {A}\rightarrow \mathfrak {H}_{\mathcal {A},\mathcal {J}},\quad \nabla (A)=(\partial _j(A))_{j\in \mathcal {J}}, \end{aligned}$$

which provide analogs of the partial derivatives and the usual gradient operator, respectively. The commutator $\partial _j$ satisfies the product rule

1 $$\begin{aligned} \partial _j(AB)=A\partial _j (B)+\partial _j(A)B. \end{aligned}$$

Note that in contrast too the usual partial derivatives, the order of the factors plays a role here. This is one central reason for many of the differences and intricacies of the quantum optimal transport distance compared to the classical Wasserstein distance.

Continuing with the analogy with calculus, we write ${\text {div}}$ for the adjoint of $-\nabla$, that is,

$$\begin{aligned} {\text {div}}=-\sum _{j\in \mathcal {J}}\partial _j^\dagger . \end{aligned}$$

The crucial ingredient in the definition of $\mathcal {W}$, which allows to deal with the noncommutativity of the product rule, is the operator $[\rho ]_{\varvec{\omega }}$, whose definition we recall next. For $X\in \mathcal {A}_+$ and $\alpha \in \mathbb {R}$ define

$$\begin{aligned}{}[X]_\alpha :\mathfrak {H}_\mathcal {A}\rightarrow \mathfrak {H}_\mathcal {A},\quad [X]_\alpha (A)=\int _0^1 e^{\alpha (s-1/2)}X^s A X^{1-s}\,ds. \end{aligned}$$

The motivation for this definition is a chain rule identity [8,  Eq. (5.7)], which can best be illustrated in the case $\alpha =0$:

$$\begin{aligned} {[}X]_0(\partial _j(\log X))=\partial _j(X). \end{aligned}$$

Given $\varvec{\alpha }=(\alpha _j)_{j\in \mathcal {J}}$, we define

$$\begin{aligned} {[}X]_{\varvec{\alpha }}:\mathfrak {H}_{\mathcal {A},\mathcal {J}}\rightarrow \mathfrak {H}_{\mathcal {A},\mathcal {J}},\quad (\mathbf {V}_j)_{j\in \mathcal {J}}\mapsto ([X]_{\alpha _j}\mathbf {V}_j)_{j\in \mathcal {J}}. \end{aligned}$$

For $\varepsilon \ge 0$ we write  for the set of all pairs $(\rho ,\mathbf {V})$ such that $\rho \in H^1([0,1];\mathfrak {S}_+(\mathcal {A}))$ with $\rho (0)=\rho _0$, $\rho (1)=\rho _1$, $\mathbf {V}\in L^2([0,1];\mathfrak {H}_{\mathcal {A},\mathcal {J}})$ and

2 $$\begin{aligned} \dot{\rho }(t) +{\text {div}}\mathbf {V}(t)=\varepsilon \mathscr {L}^\dagger \rho (t) \end{aligned}$$

for a.e. $$t\in [0,1]$$.

Here and in the following we write $H^1([0,1];\mathfrak {S}_+(\mathcal {A}))$ for the space of all maps $\rho :[0,1]\rightarrow \mathfrak {S}_+(\mathcal {A})$ such that $(t\mapsto \tau (A\rho (t)))\in H^1([0,1])$ for all $A\in \mathcal {A}$. The space $L^2([0,1];\mathfrak {H}_{\mathcal {A},\mathcal {J}})$ and other vector-valued functions spaces occurring later are defined similarly.

We define a metric $\mathcal {W}_\varepsilon$ on $\mathfrak {S}_+(\mathcal {A})$ bywhere $\varvec{\omega }=(\omega _j)_{j\in \mathcal {J}}$ with the Bohr frequencies $\omega _j$ of $\mathscr {L}$.

For $\varepsilon =0$, this is the noncommutative transport distance $\mathcal {W}$ introduced in [8] (as distance function associated with a Riemannian metric on $\mathfrak {S}(\mathcal {A})_+$), and for $\varepsilon >0$, this is the entropic regularization of $\mathcal {W}$ introduced in [3].

A standard mollification argument shows that the infimum in the definition of $\mathcal {W}_\varepsilon$ can equivalently be taken over  with $\rho \in C^\infty ([0,1];\mathfrak {S}_+(\mathcal {A}))$. More precisely, if  and $(\eta _\delta )_{\delta >0}$ is a mollifying kernel, then $(\rho *\eta _\delta ,\mathbf {V}*\eta _\delta )$ satisfies (2). A suitable reparametrization of the time parameter gives a pair  such that $\rho ^\delta$ is smooth and

$$\begin{aligned} \lim _{\delta \searrow 0}\int _0^1\langle \mathbf {V}^\delta (t),[\rho ^\delta (t)]_{\varvec{\omega }}^{-1}\mathbf {V}^\delta (t)\rangle \,dt=\int _0^1\langle \mathbf {V}(t),[\rho (t)]_{\varvec{\omega }}^{-1}\mathbf {V}(t)\rangle \,dt \end{aligned}$$

By a substitution one can reformulate the minimization problem for $\mathcal {W}_\varepsilon$ in such a way that the constraint becomes independent from $\varepsilon$. For that purpose define the relative entropy of $\rho \in \mathfrak {S}_+(\mathcal {A})$ with respect to $\sigma$ by

$$\begin{aligned} D(\rho \Vert \sigma )=\tau (\rho (\log \rho -\log \sigma )) \end{aligned}$$

and the Fisher information of $\rho \in \mathfrak {S}_+(\mathcal {A})$ by

$$\begin{aligned} \mathcal {I}(\rho )=\langle [\rho ]_{\varvec{\omega }}\nabla (\log \rho -\log \sigma ),\nabla (\log \rho -\log \sigma )\rangle _{\mathfrak {H}_{\mathcal {A},\mathcal {J}}}. \end{aligned}$$

According to [3,  Theorem 1], one hasThe metric $\mathcal {W}$ is intimately connected to the relative entropy and therefore well-suited to study its decay properties along the QMS. For other applications, variants of the metric $\mathcal {W}$ have also proven useful (e.g. [10, 11]), for which the operator $[\rho ]_{\varvec{\omega }}$ is replaced. A systematic framework of these metrics has been developed in [9, 30]. It can be conveniently phrased in terms of so-called operator connections.

Let *H* be an infinite-dimensional Hilbert space. A map $\Lambda :B(H)_+\times B(H)_+\rightarrow B(H)_+$ is called an *operator connection* [21] if

- $A\le C$ and $B\le D$ imply $\Lambda (A,B)\le \Lambda (C,D)$ for $A,B,C,D\in B(H)_+$,
- $$C\Lambda (A,B)C\le \Lambda (CAC,CBC)$$ for $$A,B,C\in B(H)_+$$,
- $$A_n\searrow A$$, $$B_n\searrow B$$ imply $$\Lambda (A_n,B_n)\searrow \Lambda (A,B)$$ for $$A,A_n,B,B_n\in B(H)_+$$.

For example, for every $\alpha \in \mathbb {R}$ the map

$$\begin{aligned} \Lambda _\alpha :(A,B)\mapsto \int _0^1 e^{\alpha (s-1/2)}A^s B^{1-s}\,ds \end{aligned}$$

is an operator connection.

It can be shown that every operator connection $\Lambda$ satisfies

$$\begin{aligned} U^*\Lambda (A,B)U=\Lambda (U^*AU,U^*BU) \end{aligned}$$

for $A,B\in B(H)_+$ and unitary $U\in B(H)$ [21,  Sect. 2]. Embedding $\mathbb {C}^n$ into *H*, one can view $A,B\in M_n(\mathbb {C})$ as bounded linear operators on *H*, and the unitary invariance of $\Lambda$ ensures that $\Lambda (A,B)$ does not depend on the embedding of $\mathbb {C}^n$ into *H*.

For $$X\in \mathcal {A}$$ define

$$\begin{aligned}&L(X):\mathfrak {H}_{\mathcal {A}}\rightarrow \mathfrak {H}_{\mathcal {A}},\quad A\mapsto XA\\&R(X):\mathfrak {H}_{\mathcal {A}}\rightarrow \mathfrak {H}_{\mathcal {A}},\quad A\mapsto AX. \end{aligned}$$

Note that if $X\in \mathcal {A}_+$, then

$$\begin{aligned} \langle A,L(X) A\rangle _{\mathfrak {H}_\mathcal {A}}=\tau (A^*X A)=\tau ((X^{1/2}A)^*(X^{1/2}A))\ge 0, \end{aligned}$$

so that *L*(*X*) is a positive operator, and the same holds for *R*(*X*).

Thus we can define

$$\begin{aligned} {[}X]_\Lambda =\Lambda (L(X),R(X)). \end{aligned}$$

If $\lambda ,\mu \ge 0$ and $1_n$ denotes the identity matrix, then $\Lambda (\lambda 1_n,\mu 1_n)$ is a scalar multiple of the identity as a consequence the unitary invariance of $\Lambda$ discussed above. By a slight abuse of notation, this scalar will be denoted by $\Lambda (\lambda ,\mu )$.

Since *L*(*X*) and *R*(*X*) commute, we have

3 $$\begin{aligned} \Lambda (L(X),R(X))A=\sum _{k,l=1}^n \Lambda (\lambda _k,\lambda _l)E_k A E_l \end{aligned}$$

for $X\in \mathcal {A}_+$ and $A\in \mathfrak {H}_\mathcal {A}$, where $(\lambda _k)$ are the eigenvalues of *X* and $E_k$ the corresponding spectral projections.

More generally let $\varvec{\Lambda }=(\Lambda _j)_{j\in \mathcal {J}}$ be a family of operator connections and define

$$\begin{aligned}&{[}\rho ]_{\Lambda _j}=\Lambda _j(L(\rho ),R(\rho )),\\&{[}\rho ]_{\varvec{\Lambda }}=\bigoplus _{j\in \mathcal {J}}[\rho ]_{\Lambda _j}. \end{aligned}$$

Clearly, $[\rho ]_\alpha =[\rho ]_{\Lambda _{\alpha }}$ with the operator connection $\Lambda _\alpha$ from above.

Then one can define a distance $\mathcal {W}_{\varvec{\Lambda },\varepsilon }$ byIf $\Lambda _j=\Lambda _{\omega _j}$ as above, then we retain the original metric $\mathcal {W}_\varepsilon$, while for $\Lambda _j(A,B)=\frac{1}{2} (A+B)$ (and $\varepsilon =0$) one obtains the distance studied in [10, 11].

Later we will make the additional assumption that $\Lambda _{j^*}(A,B)=\Lambda _j(B,A)$, where $j^*\in \mathcal {J}$ is the unique index in the Alicki representation of $\mathscr {L}$ such that $V_{j^*}=V_j^*$. It follows from the representation theorem of operator means [21] that the class of metrics $\mathcal {W}_{\varvec{\Lambda },0}$ with $\varvec{\Lambda }$ subject to this symmetry condition is exactly the class of metrics satisfying Assumptions 7.2 and 9.5 in [9].

For technical reasons in the proof of Theorem 2, it will be necessary to allow for curves of density matrices that are not necessarily invertible. For this purpose, we make the following convention: If $\mathcal {K}:\mathfrak {H}_{\mathcal {A},\mathcal {J}}\rightarrow \mathfrak {H}_{\mathcal {A},\mathcal {J}}$ is a positive operator and $\mathbf {V}\in \mathfrak {H}_{\mathcal {A},\mathcal {J}}$, we define

$$\begin{aligned} \langle \mathbf {V},\mathcal {K}^{-1}\mathbf {V}\rangle _{\mathfrak {H}_{\mathcal {A},\mathcal {J}}}={\left\{ \begin{array}{ll} \langle \mathcal {K}\mathbf {W},\mathbf {W}\rangle _{\mathfrak {H}_{\mathcal {A},\mathcal {J}}}&{}\text {if }\mathbf {V}\in (\ker \mathcal {K})^\perp ,\mathcal {K}\mathbf {W}=\mathbf {V},\\ \infty &{}\text {otherwise}.\end{array}\right. } \end{aligned}$$

Since $(\ker \mathcal {K})^\perp ={\text {ran}}\mathcal {K}$ and $\mathcal {K}$ is injective on $(\ker \mathcal {K})^\perp$, the element $\mathbf {W}$ in this definition exists and is unique. Moreover, this convention is clearly consistent with the usual definition if $\mathcal {K}$ is invertible.

Alternatively, as a direct consequence of the spectral theorem, this expression can equivalently be defined as

$$\begin{aligned} \langle \mathbf {V},\mathcal {K}^{-1}\mathbf {V}\rangle _{\mathfrak {H}_{\mathcal {A},\mathcal {J}}}=\sum _{k=1}^m \frac{1}{\lambda _k}|\langle \mathbf {V},\mathbf {W}_k\rangle _{\mathfrak {H}_{\mathcal {A},\mathcal {J}}}|^2, \end{aligned}$$

where $\lambda _1,\dots ,\lambda _m$ are the eigenvalues of $\mathcal {K}$ and $\mathcal {W}_1,\dots ,W_m$ an orthonormal basis of corresponding eigenvectors.

### Lemma 1

If $\mathcal K_n:\mathfrak {H}_{\mathcal {A},\mathcal {J}}\rightarrow \mathfrak {H}_{\mathcal {A},\mathcal {J}},$ $n\in \mathbb N,$ are positive invertible operators that converge monotonically decreasing to $\mathcal {K},$ then

$$\begin{aligned} \langle \mathbf {V},\mathcal K_n^{-1}\mathbf {V}\rangle _{\mathfrak {H}_{\mathcal {A},\mathcal {J}}}\nearrow \langle \langle \mathbf {V},\mathcal K^{-1}\mathbf {V}\rangle _{\mathfrak {H}_{\mathcal {A},\mathcal {J}}} \end{aligned}$$

for all $$\mathbf {V}\in \mathfrak {H}_{\mathcal {A},\mathcal {J}}$$.

### Proof

From the spectral expression it is easy to see that

$$\begin{aligned} \langle \mathbf {V},\mathcal {K}_n^{-1}\mathbf {V}\rangle _{\mathfrak {H}_{\mathcal {A},\mathcal {J}}}=\sup _{\delta >0}\langle \mathbf {V},(\mathcal {K}_n+\delta )^{-1}\mathbf {V}\rangle _{\mathfrak {H}_{\mathcal {A},\mathcal {J}}} \end{aligned}$$

and the same for $\mathcal {K}_n$ replaced by $\mathcal {K}$. Moreover, since $\mathcal {K}_n\searrow \mathcal {K}$, we have $(\mathcal {K}_n+\delta )^{-1}\nearrow (\mathcal {K}+\delta )^{-1}$. Thus

$$\begin{aligned} \langle \mathbf {V},\mathcal {K}^{-1}\mathbf {V}\rangle _{\mathfrak {H}_{\mathcal {A},\mathcal {J}}}&=\sup _{\delta>0} \langle \mathbf {V},(\mathcal {K}+\delta )^{-1}\mathbf {V}\rangle _{\mathfrak {H}_{\mathcal {A},\mathcal {J}}}\\&=\sup _{\delta>0}\sup _{n\in \mathbb N}\langle \mathbf {V},(\mathcal {K}_n+\delta )^{-1} \mathbf {V}\rangle _{\mathfrak {H}_{\mathcal {A},\mathcal {J}}}\\&=\sup _{n\in \mathbb N}\sup _{\delta >0}\langle \mathbf {V},(\mathcal {K}_n+\delta )^{-1} \mathbf {V}\rangle _{\mathfrak {H}_{\mathcal {A},\mathcal {J}}}\\&=\sup _{n\in \mathbb N}\langle \mathbf {V},\mathcal {K}_n^{-1}\mathbf {V}\rangle _{\mathfrak {H}_{\mathcal {A},\mathcal {J}}}. \end{aligned}$$

Since $(\langle \mathbf {V},\mathcal {K}_n^{-1}\mathbf {V}\rangle _{\mathfrak {H}_{\mathcal {A},\mathcal {J}}})$ is monotonically increasing, this settles the claim.

Write  for the set of all pairs $(\rho ,\mathbf {V})$ such that $\rho \in H^1([0,1];\mathfrak {S}(\mathcal {A}))$ with $\rho (0)=\rho _0$, $\rho (1)=\rho _1$, $\mathbf {V}\in L^2([0,1];\mathfrak {H}_{\mathcal {A},\mathcal {J}})$ and

$$\begin{aligned} \dot{\rho }(t)+{\text {div}}\mathbf {V}(t)=\varepsilon \mathscr {L}^\dagger \rho (t) \end{aligned}$$

for a.e. $t\in [0,1]$. The only difference to the definition of  is that $\rho (t)$ is not assumed to be invertible.

### Proposition 1

For $\rho _0,\rho _1\in \mathfrak {S}_+(\mathcal {A})$ we have

### Proof

It suffices to show that every curve  can be approximated by curves in  such that the action integrals converge.

For that purpose let

$$\begin{aligned}&\rho ^\delta :[0,1]\rightarrow \mathfrak {S}_+(\mathcal {A}),\\&t\mapsto {\left\{ \begin{array}{ll}(1-t)\rho _0+t1_\mathcal {A}&{}\text {if }t\in [0,\delta ],\\ (1-\delta )\rho ((1-2\delta )^{-1}(t-\delta ))+\delta 1_\mathcal {A}&{}\text {if }t\in (\delta ,1-\delta ),\\ t\rho _1+(1-t)1_\mathcal {A}&{}\text {if }t\in [1-\delta ,1]. \end{array}\right. } \end{aligned}$$

Since $(P_t)$ is assumed to be ergodic, by [8,  Theorem 5.4] there exists for every $t\in [0,1]$ a unique $X(t)\in \mathcal {A}_h$ with $\tau (X(t)))=0$ such that

$$\begin{aligned} 1_\mathcal {A}-\rho _0+{\text {div}}(\nabla X(t))=\varepsilon (1-t)\mathscr {L}^\dagger \rho _0, \end{aligned}$$

and *X*(*t*) depends continuously on *t*. For $t\in [0,\delta ]$ let $\mathbf {V}^\delta (t)=\nabla X(t)$.

Moreover, if $\lambda$ is the smallest eigenvalue of $\rho _0$, which is strictly positive by assumption, then $\rho ^\delta (t)\ge ((1-t)\lambda +t)1_\mathcal {A}\ge \lambda 1_\mathcal {A}$.

Thus

$$\begin{aligned} \int _0^\delta \langle \mathbf {V}^\delta (t),[\rho ^\delta (t)]_{\varvec{\Lambda }}^{-1} \mathbf {V}^\delta (t)\rangle _{\mathfrak {H}_{\mathcal {A},\mathcal {J}}}\,dt&\le \int _0^\delta \langle \mathbf {V}^\delta (t),[\lambda 1_\mathcal {A}]_{\varvec{\Lambda }}^{-1}\mathbf {V}^\delta (t)\rangle _{\mathfrak {H}_{\mathcal {A},\mathcal {J}}}\,dt\\&\le \Vert [\lambda 1_\mathcal {A}]_{\varvec{ \Lambda }}^{-1}\Vert \int _0^\delta \Vert \nabla X(t)\Vert _{\mathfrak {H}_{\mathcal {A},\mathcal {J}}}^2\,dt\\&\rightarrow 0 \end{aligned}$$

as $\delta \rightarrow 0$. Similarly one can show

$$\begin{aligned} \lim _{\delta \rightarrow 0}\int _{1-\delta }^1 \langle \mathbf {V}^\delta (t),[\rho ^\delta (t)]_{\varvec{\Lambda }}^{-1}\mathbf {V}^\delta (t)\rangle _{\mathfrak {H}_{\mathcal {A},\mathcal {J}}}\,dt=0. \end{aligned}$$

By the same argument as above, for a.e. $t\in (\delta ,1-\delta )$ there exists a unique gradient $\mathbf {W}^\delta (t)$ such that

$$\begin{aligned} {\text {div}}\mathbf {W}^\delta (t)=-\frac{2\delta \varepsilon }{1-2\delta }\mathscr {L}^\dagger \rho ((1-2\delta )^{-1}(t-\delta )) \end{aligned}$$

and

$$\begin{aligned} \Vert \mathbf {W}^\delta (t)\Vert _{\mathfrak {H}_{\mathcal {A},\mathcal {J}}}\le \frac{2\delta \varepsilon }{1-2\delta }\Vert \mathscr {L}^\dagger \rho ((1-2\delta )^{-1} (t-\delta ))\Vert _{\mathfrak {H}_{\mathcal {A},\mathcal {J}}}. \end{aligned}$$

Since $\rho \in H^1([0,1];\mathfrak {S}(\mathcal {A}))\subset C([0,1];\mathfrak {S}(\mathcal {A}))$, the norm on the right side is bounded independent of $\delta$, so that

$$\begin{aligned} \Vert \mathbf {W}^\delta (t)\Vert _{\mathfrak {H}_{\mathcal {A},\mathcal {J}}}\le \tilde{C}\delta \end{aligned}$$

with a constant $\tilde{C}>0$ independent of $\delta$. As $\rho ^\delta (t)\ge \delta 1_\mathcal {A}$ for $t\in (\delta ,1-\delta )$, this implies

$$\begin{aligned} \int _\delta ^{1-\delta }\langle \mathbf {W}^\delta (t), [\rho ^\delta (t)]_{\varvec{\Lambda }}^{-1} \mathbf {W}^\delta (t)\rangle _{\mathfrak {H}_{\mathcal {A},\mathcal {J}}}\,dt&\le \frac{1}{\delta }\int _\delta ^{1-\delta } \langle \mathbf {W}^\delta (t),[1_\mathcal {A}]_{\varvec{\Lambda }}^{-1}\mathbf {W}^\delta (t)\rangle _{\mathfrak {H}_{\mathcal {A},\mathcal {J}}}\,dt\\&\le \tilde{C} \Vert [1_\mathcal {A}]_{\varvec{\Lambda }}^{-1}\Vert \delta \\&\rightarrow 0 \end{aligned}$$

as $$\delta \rightarrow 0$$.

With

$$\begin{aligned} \mathbf {V}^\delta (t)=\frac{1}{1-2\delta }\mathbf {V}((1-2\delta )^{-1}(t-\delta ))+\mathbf {W}^\delta (t) \end{aligned}$$

we have

$$\begin{aligned} \dot{\rho }^\delta (t)+{\text {div}}\mathbf {V}^\delta (t)=\varepsilon \mathscr {L}\rho ^\delta (t). \end{aligned}$$

Furthermore,

$$\begin{aligned}&\int _\delta ^{1-\delta }\left\langle \mathbf {V}\left( \frac{t-\delta }{1-2\delta }\right) ,[\rho ^\delta (t)]_{\varvec{\Lambda }}^{-1} \mathbf {V}\left( \frac{t-\delta }{1-2\delta }\right) \right\rangle _{\mathfrak {H}_{\mathcal {A},\mathcal {J}}}\,dt\\&\quad =\frac{1-2\delta }{1-\delta }\int _0^1 \left\langle \mathbf {V}(s),\left[ \rho (s)+\frac{\delta }{1-\delta }\right] _{\varvec{\Lambda }}^{-1}\mathbf {V}(s)\right\rangle _{\mathfrak {H}_{\mathcal {A},\mathcal {J}}}\,ds, \end{aligned}$$

where we used the substitution $s=(1-2\delta )^{-1}(t-\delta )$.

By Lemma 1 and the monotone convergence theorem we obtain

$$\begin{aligned}&\lim _{\delta \rightarrow 0}\int _\delta ^{1-\delta }\left\langle \mathbf {V}\left( \frac{t-\delta }{1-2\delta }\right) ,[\rho ^\delta (t)]_{\varvec{\Lambda }}^{-1} \mathbf {V}\left( \frac{t-\delta }{1-2\delta }\right) \right\rangle _{\mathfrak {H}_{\mathcal {A},\mathcal {J}}}\,dt\\&\quad =\int _0^1 \langle \mathbf {V}(s),[\rho (s)]_{\varvec{\Lambda }}^{-1}\mathbf {V}(s)\rangle _{\mathfrak {H}_{\mathcal {A},\mathcal {J}}}\,ds. \end{aligned}$$

Together with the convergence result for $\mathbf {W}^\delta$ from above, this implies

$$\begin{aligned} \int _\delta ^{1-\delta }\langle \mathbf {V}^\delta (t),[\rho ^\delta (t)]_{\varvec{\Lambda }}^{-1}\mathbf {V}^\delta (t)\rangle _{\mathfrak {H}_{\mathcal {A},\mathcal {J}}}\,dt\rightarrow \int _0^1\langle \mathbf {V}(t),[\rho (t)]_{\varvec{\Lambda }}^{-1}\mathbf {V}(t)\rangle _{\mathfrak {H}_{\mathcal {A},\mathcal {J}}}\,dt. \end{aligned}$$

Altogether we have shown

$$\begin{aligned} \lim _{\delta \rightarrow 0}\int _0^{1}\langle \mathbf {V}^\delta (t),[\rho ^\delta (t)]_{\varvec{\Lambda }}^{-1}\mathbf {V}^\delta (t)\rangle _{\mathfrak {H}_{\mathcal {A},\mathcal {J}}}\,dt= \int _0^1\langle \mathbf {V}(t),[\rho (t)]_{\varvec{\Lambda }}^{-1}\mathbf {V}(t)\rangle _{\mathfrak {H}_{\mathcal {A},\mathcal {J}}}\,dt. \end{aligned}$$

$$\square $$

## Real subspaces

Since the proof of the main result relies on convex analysis methods for real Banach spaces, we need to identify suitable real subspaces for our purposes. For $\mathcal {A}$ this is simply $\mathcal {A}_h$, but for $\mathfrak {H}_{\mathcal {A},\mathcal {J}}$ this is less obvious and will be done in the following.

For $j\in \mathcal {J}$ denote by $j^*$ the unique index in $\mathcal {J}$ such that $V_j^*=V_{j^*}$. Let $\tilde{\mathfrak {H}}_ {\mathcal {A}}^{(j)}$ be the linear span of $\{X\partial _j A\mid A,X\in \mathcal {A}\}$, and define a linear map $J:\tilde{\mathfrak {H}}_{\mathcal {A}}^{(j)}\rightarrow \tilde{\mathfrak {H}}_{\mathcal {A}}^{(j^*)}$ by

$$\begin{aligned} J(X\partial _j A)=\partial _{j^*} (A^*)X^*. \end{aligned}$$

By the product rule (1), $(\partial _j A)X$ also belongs to $\tilde{\mathfrak {H}}_{\mathcal {A},\mathcal {J}}^{(j)}$ and

$$\begin{aligned} J((\partial _j A)X)=X^*\partial _{j^*}(A^*). \end{aligned}$$

Thus *J* interchanges left and right multiplication, that is, $J(A\mathbf {V}B)=B^*J(\mathbf {V}) A^*$ for $A,B\in \mathcal {A}$ and $\mathbf {V}\in \tilde{\mathfrak {H}}_\mathcal {A}^{(j)}$.

### Lemma 2

The map *J* is anti-unitary.

### Proof

For $A,B,X,Y\in \mathcal {A}$ we have

$$\begin{aligned} \langle J(X\partial _j A),J(Y\partial _j B)\rangle _{\mathfrak {H}_\mathcal {A}}&= \tau (X(AV_j-V_j A)(V_j^*B^*-B^*V_j^*) Y^*)\\&=\tau ((B^*V_j^*-V_j^*B^*)Y^*X(V_j A-AV_j))\\&=\langle Y\partial _j B,X\partial _j A\rangle . \end{aligned}$$

$$\square $$

Let

$$\begin{aligned} \mathcal {H}^{(h)}_{\mathcal {A},\mathcal {J}}=\left\{ \mathbf {V}\in \bigoplus _{j\in \mathcal {J}}\tilde{\mathfrak {H}}_{\mathcal {A}}^{(j)}\mid J(\mathbf {V}_j)=\mathbf {V}_{j^*}\right\} . \end{aligned}$$

By the previous lemma, $\mathcal {H}^{(h)}_{\mathcal {J},\mathcal {A}}$ is a real Hilbert space.

### Lemma 3

Let $(\Lambda _j)_{j\in \mathcal {J}}$ be a family of operator connections such that

$$\begin{aligned} \Lambda _{j}(B,A)=\Lambda _{j^*}(A,B) \end{aligned}$$

for all $j\in \mathcal {J}$. If $A\in \mathcal {A}_h$ and $\rho \in \mathfrak {S}(\mathcal {A}),$ then $\nabla A,[\rho ]_{\varvec{\Lambda }}\nabla A\in \mathcal {H}^{(h)}_{\mathcal {A},\mathcal {J}}$.

### Proof

For $\nabla A$ the statement follows directly from the definitions. For $[\rho ]_{\varvec{\Lambda }} \nabla A$ first note that

$$\begin{aligned} J\Lambda (L(\rho ),R(\rho ))=\Lambda (R(\rho ),L(\rho ))J \end{aligned}$$

as a consequence of the spectral representation (3) and the fact that *J* interchanges left and right multiplication.

Thus

$$\begin{aligned} J([\rho ]_{\Lambda _j}\partial _j A)&=J\Lambda _j(L(\rho ),R(\rho ))\partial _j A\\&=\Lambda _j(R(\rho ),L(\rho ))J\partial _j A\\&=\Lambda _{j^*}(L(\rho ),R(\rho ))\partial _{j^*}A. \end{aligned}$$

$$\square $$

## Duality

In this section we prove the duality theorem announced in the introduction. Our strategy follows the same lines as the proof in the commutative case in [15]. It crucially relies on the Rockafellar–Fenchel duality theorem quoted below. Throughout this section we fix an ergodic QMS with generator $\mathscr {L}$ satisfying the $\sigma$-DBC for some $\sigma \in \mathfrak {S}_+(\mathcal {A})$ and a family $(\Lambda _j)_{j\in \mathcal {J}}$ of operator connections such that $\Lambda _{j^*}(A,B)=\Lambda _j(B,A)$ for all $j\in \mathcal {J}$.

We need the following definition for the constraint of the dual problem. Here and in the following we write

$$\begin{aligned} \langle \mathbf {V},\mathbf {W}\rangle _\rho =\langle \mathbf {V},[\rho ]_{\varvec{\Lambda }}\mathbf {W}\rangle _{\mathfrak {H}_{\mathcal {A},\mathcal {J}}} \end{aligned}$$

for $$\mathbf {V},\mathbf {W}\in \mathfrak {H}_{\mathcal {A},\mathcal {J}}$$ and $$\rho \in \mathcal {A}_+$$.

### Definition 1

A function $A\in H^1((0,T);\mathcal {A}_h)$ is said to be a *Hamilton–Jacobi–Bellmann subsolution* if for a.e. $t\in (0,T)$ we have

$$\begin{aligned} \tau ((\dot{A}(t)+\varepsilon \mathscr {L}A(t)) \rho )+\frac{1}{2} \Vert \nabla A(t)\Vert _{\rho }^2\le 0\quad \text {for all }\rho \in \mathfrak {S}(A). \end{aligned}$$

The set of all Hamilton–Jacobi–Bellmann subsolutions is denoted by $\mathsf {HJB}_{\varvec{\Lambda ,\varepsilon }}$.

Our proof will establish equality between the primal and dual problem, but before we begin, let us show that one inequality is actually quite easy to obtain.

### Proposition 2

For all $\rho _0,\rho _1\in \mathfrak {S}_+(\mathcal {A})$ we have

### Proof

For $A\in \mathsf {HJB}_{\varvec{\Lambda ,\varepsilon }}$ and  we have

$$\begin{aligned} \tau (A(1)\rho _1-A(0)\rho _0)&=\int _0^1 \tau (\dot{A}(t) \rho (t)+A(t)\dot{\rho }(t))\,dt\\&\le -\int _0^1\left( \varepsilon \tau ((\mathscr {L}A(t))\rho (t))+\frac{1}{2}\Vert \nabla A(t)\Vert _{\rho (t)}^2\right) \,dt\\&\quad +\int _0^1(\langle \nabla A(t),\mathbf {V}(t)\rangle _{\mathfrak {H}_{\mathcal {A},\mathcal {J}}}+\varepsilon \tau (A(t)\mathscr {L}^\dagger \rho (t)))\,dt\\&= \int _0^1\langle [\rho (t)]_{\varvec{\Lambda }}^{1/2}\nabla A(t),[\rho (t)]_{\varvec{\Lambda }}^{-1/2}\mathbf {V}(t)\rangle _{\mathfrak {H}_{\mathcal {A},\mathcal {J}}}\,dt\\&\quad -\frac{1}{2} \int _0^1\langle [\rho (t)]_{\varvec{\Lambda }}^{1/2}\nabla A(t),[\rho (t)]_{\varvec{\Lambda }}^{1/2}\nabla A(t)\rangle _{\mathfrak {H}_{\mathcal {A},\mathcal {J}}}\,dt\\&\le \frac{1}{2}\int _0^1 \langle [\rho (t)]_{\varvec{\Lambda }}^{1/2}\nabla A(t),[\rho (t)]_{\varvec{\Lambda }}^{1/2}\nabla A(t)\rangle _{\mathfrak {H}_{\mathcal {A},\mathcal {J}}}^2\,dt\\&\quad +\frac{1}{2} \int _0^1\langle [\rho (t)]_{\varvec{\Lambda }}^{-1/2}\nabla A(t),[\rho (t)]_{\varvec{\Lambda }}^{-1/2}\nabla A(t)\rangle _{\mathfrak {H}_{\mathcal {A},\mathcal {J}}}^2\,dt\\&\quad -\frac{1}{2} \int _0^1\langle [\rho (t)]_{\varvec{\Lambda }}^{1/2}\nabla A(t),[\rho (t)]_{\varvec{\Lambda }}^{1/2}\nabla A(t)\rangle _{\mathfrak {H}_{\mathcal {A},\mathcal {J}}}\,dt\\&=\frac{1}{2}\int _0^1\langle \mathbf {V}(t),[\rho (t)]_{\varvec{\Lambda }}^{-1}\mathbf {V}(t)\rangle _{\mathfrak {H}_{\mathcal {A},\mathcal {J}}}\,dt, \end{aligned}$$

where we used $A\in \mathsf {HJB}_{\varvec{\Lambda ,\varepsilon }}$ and  for the first inequality and Young’s inequality $2|\langle \xi ,\eta \rangle |\le \langle \xi ,\xi \rangle +\langle \eta ,\eta \rangle$ for the second inequality.$\square$

To prove actual equality, our crucial tool is the Rockafellar–Fenchel duality theorem (see e.g. [28,  Theorem 1.9], which we quote here for the convenience of the reader. Recall that if *E* is a (real) normed space, the Legendre–Fenchel transform $F^*$ of a proper convex function $F:E\rightarrow \mathbb {R}\cup \{\infty \}$ is defined by

$$\begin{aligned} F^*:E^*\rightarrow \mathbb {R}\cup \{\infty \},\,F^*(x^*)=\sup _{x\in E}(\langle x^*,x\rangle -F(x)). \end{aligned}$$

### Theorem 1

Let *E* be a real normed space and $F,G:E\rightarrow \mathbb {R}\cup \{\infty \}$ proper convex functions with Legendre–Fenchel transforms $F^*,G^*$. If there exists $z_0\in E$ such that *G* is continuous at $z_0$ and $F(z_0),G(z_0)<\infty ,$ then

$$\begin{aligned} \sup _{z\in E}(-F(z)-G(z))=\min _{z^*\in E^*}(F^*(z^*)+G^*(-z^*)). \end{aligned}$$

Before we state the main result, we still need the following useful inequality.

### Lemma 4

For any operator connection $\Lambda$ the map

$$\begin{aligned} f_{\Lambda }:\mathcal {A}_{++}\rightarrow B(\mathfrak {H}_A),\,A\mapsto [A]_\Lambda \end{aligned}$$

is smooth and its Fréchet derivative satisfies

$$\begin{aligned} d f_\Lambda (B) A\ge f_\Lambda (A) \end{aligned}$$

for $A,B\in \mathcal {A}_{++}$ with equality if $A=B$.

### Proof

Smoothness of $f_\Lambda$ is a consequence of the representation theorem of operator connections [21,  Theorem 3.4]. For the claim about the Fréchet derivative first note that $f_\Lambda$ is concave [21,  Theorem 3.5]. Therefore $d^2 f_\Lambda (X)[Y,Y]\le 0$ for all $X\in \mathcal {A}_{++}$ and $Y\in \mathcal {A}_h$ by [18,  Proposition 2.2].

The fundamental theorem of calculus implies

$$\begin{aligned} (d f_\Lambda (A)-d f_\Lambda (B))(A-B)&=\int _0^1 d^2 f_\Lambda (tA+(1-t)B)[A-B,A-B]\,dt\\&\le 0. \end{aligned}$$

Since $f_\Lambda$ is 1-homogeneous by [21,  Eq. (2.1)], its derivative is 0-homogeneous. Thus, if we replace *B* by $\varepsilon B$ and let $\varepsilon \searrow 0$, we obtain

$$\begin{aligned} d f_\Lambda (A)A\le d f_\Lambda (B)A. \end{aligned}$$

Moreover, the 1-homogeneity of $f_\Lambda$ implies $d f_\Lambda (A)A=f_\Lambda (A)$, which settles the claim.

### Theorem 2

(Duality formula) For $\rho _0,\rho _1\in \mathfrak {S}_+(A)$ we have

$$\begin{aligned} \frac{1}{2}\mathcal {W}_{\varvec{\Lambda ,\varepsilon }}(\rho _0,\rho _1)^2&= \sup \{\tau (A(1)\rho _1)-\tau (A(0)\rho _0) : A\in \mathsf {HJB}_{\varvec{\Lambda ,\varepsilon }}\}\\&=\sup \{\tau (A(1)\rho _1)-\tau (A(0)\rho _0) : A\in \mathsf {HJB}_{\varvec{\Lambda ,\varepsilon }}\cap C^\infty ([0,1];\mathcal {A})\}. \end{aligned}$$

### Proof

The second inequality follows easily by mollifying. We will show the duality formula for Hamilton–Jacobi subsolutions in $H^1$. For this purpose we use the Rockafellar–Fenchel duality formula from Theorem 1.

Let *E* be the real Banach space

$$\begin{aligned} H^1([0,1];\mathcal {H}^{(h)}_\mathcal {A})\times L^2([0,1];\mathcal {H}^{(h)}_{\mathcal {A},\mathcal {J}}). \end{aligned}$$

By the theory of linear ordinary differential equations, the map

$$\begin{aligned} H^1([0,1];\mathcal {H}^{(h)}_\mathcal {A})\rightarrow \mathcal {H}^{(h)}_\mathcal {A}\times L^2([0,1];\mathcal {H}^{(h)}_\mathcal {A}),\quad A\mapsto (A(0),\dot{A}+\varepsilon \mathscr {L}A) \end{aligned}$$

is a linear isomorphism.

Thus the dual space $E^*$ can be isomorphically identified with

$$\begin{aligned} \mathcal {H}^{(h)}_\mathcal {A}\times L^2([0,1];\mathcal {H}^{(h)}_\mathcal {A})\times L^2([0,1];\mathcal {H}^{(h)}_{\mathcal {A},\mathcal {J}}) \end{aligned}$$

via the dual pairing

$$\begin{aligned} \langle (A,\mathbf {V}),(B,C,\mathbf {W})\rangle&=\tau (A(0)B)+\int _0^1 \tau ((\dot{A}(t)+\varepsilon \mathscr {L}A(t))C(t))\,dt\\&\quad +\int _0^1 \langle \mathbf {V}(t),\mathbf {W}(t)\rangle _{\mathfrak {H}_{\mathcal {A},\mathcal {J}}}\,dt. \end{aligned}$$

Define functionals $F,G:E\longrightarrow \mathbb {R}\cup \{\infty \}$ by

$$\begin{aligned} F(A,\mathbf {V})&={\left\{ \begin{array}{ll}-\tau (A(1)\rho _1)+\tau (A(0)\rho _0)&{}\text {if }\mathbf {V}=\nabla A,\\ \infty &{}\text {otherwise}, \end{array}\right. }\\ G(A,\mathbf {V})&={\left\{ \begin{array}{ll} 0&{}\text {if }(A,\mathbf {V})\in \mathcal {D},\\ \infty &{}\text {otherwise}. \end{array}\right. } \end{aligned}$$

Here $\mathcal {D}$ denotes the set of all pairs $(A,\mathbf {V})$ such that

$$\begin{aligned} \tau ((\dot{A}(t)+\varepsilon \mathscr {L}A(t))\rho )+\frac{1}{2}\Vert \mathbf {V}(t)\Vert _{\rho }^2\le 0 \end{aligned}$$

for all $$t\in [0,1]$$, $$\rho \in \mathfrak {S}(\mathcal {A})$$.

It is easy to see that *F* and *G* are convex. Moreover, for $A_0(t)=-t 1_\mathcal {A}$ and $\mathbf {V}_0=0$ we have $\mathbf {V}_0=\nabla A_0$, hence $F(A_0,\mathbf {V}_0)=0$, and

$$\begin{aligned} \tau ((\dot{A}_0(t)+\varepsilon \mathscr {L}A_0(t)) \rho )+\frac{1}{2}\Vert \mathbf {V}_0(t)\Vert _{\rho }^2=-1 \end{aligned}$$

for all $t\in [0,1],\;\rho \in \mathfrak {S}(A)$, hence $G(A_0,\mathbf {V}_0)=0$. Furthermore, *G* is clearly continuous at $(A_0,\mathbf {V}_0)$.

Moreover,

$$\begin{aligned} \sup _{(A,\mathbf {V})\in E}(-F(A,\mathbf {V})-G(A,\mathbf {V}))=\sup _{A\in \mathsf {HJB}_{\varvec{\Lambda , \varepsilon }}(\rho _0,\rho _1)}(\tau (A(1)\rho _1)-\tau (A(0)\rho _0)). \end{aligned}$$

Let us calculate the Legendre transforms of *F* and *G*, keeping in mind the identification of $E^*$. For *F* we obtain

$$\begin{aligned} F^*(B,C,\mathbf {W})&=\sup _{(A,\mathbf {V})\in E}\bigg \{\langle (A,\mathbf {V}),(B,C,\mathbf {W})\rangle -F(A,\mathbf {V})\bigg \}\\&=\sup _{A}\bigg \{\tau (A(0)B)+\int _0^1 \tau ((\dot{A} (t)+\varepsilon \mathscr {L}A(t))\,C(t))\,dt\\&\quad +\int _0^1 \langle \nabla A(t),\mathbf {W}(t)\rangle _{\mathfrak {H}_{A,\mathcal {J}}}\,dt+ \tau (A(1)\rho _1)-\tau (A(0)\rho _0)\bigg \}. \end{aligned}$$

Since the last expression is homogeneous in *A*, we have $F^*(B,C,\mathbf {W})=\infty$ unless

$$\begin{aligned} -\tau (A(1)\rho _1)+\tau (A(0)(\rho _0-B))&=\int _0^1 \tau ((\dot{A}(t)+ \varepsilon \mathscr {L}A(t))\,C(t))\,dt\\&\quad +\int _0^1 \langle \nabla A(t),\mathbf {W}(t)\rangle _{\mathfrak {H}_{\mathcal {A},\mathcal {J}}}\,dt \end{aligned}$$

for all $$A\in H^1([0,1];\mathcal {H}^{(h)}_\mathcal {A})$$.

This implies $C(0)=-(\rho _0-B)$ and $C(1)=-\rho _1$ and

$$\begin{aligned} \dot{C}(t)+{\text {div}}\mathbf {W}(t)=\varepsilon \mathscr {L}^\dagger C(t). \end{aligned}$$

ThusHere  denotes the set of all pairs $(X,\mathbf {U})\in H^1((0,1);\mathcal {H}^{(h)}_\mathcal {A})\times L^2((0,1);\mathcal {H}^{(h)}_{\mathcal {A},\mathcal {J}})$ satisfying $X(0)=\rho _0-B$, $X(1)=\rho _1$ and

$$\begin{aligned} \dot{X}(t)+{\text {div}}\varvec{U}(t)=\varepsilon \mathscr {L}^\dagger X(t). \end{aligned}$$

The difference to the definitions of  (or ) and  is that we do not make any positivity or normalization constraints. Note however that if , then

$$\begin{aligned} \frac{d}{dt}\tau (X(t))=\tau (\varepsilon \mathscr {L}^\dagger X(t)-{\text {div}}\varvec{U}(t))=0 \end{aligned}$$

so that $\tau (X(t))=\tau (\rho _1)=1$ (and $\tau (B)=0$).

Now let us turn to the Legendre transform of *G*. We have

$$\begin{aligned} G^*(B,C,\mathbf {W})&=\sup _{(A,\mathbf {V})\in E}\bigg \lbrace \langle (A,\mathbf {V}),(B,C,\mathbf {W})\rangle -G(A,\mathbf {V})\bigg \rbrace \\&=\sup _{(A,\mathbf {V})\in \mathcal {D}}\bigg \lbrace \tau (A(0)B)+\int _0^1 \tau ((\dot{A}(t)+\varepsilon \mathscr {L}A(t))C(t))\,dt\\&\quad +\int _0^1\langle \mathbf {V}(t),\mathbf {W}(t)\rangle _{\mathfrak {H}_{\mathcal {A},\mathcal {J}}})\,dt\bigg \rbrace . \end{aligned}$$

Since $(A,\mathbf {V})\in \mathcal {D}$ implies $(A+\lambda \mathrm {id}_\mathcal {A},\mathbf {V})\in \mathcal {D}$ for all $\lambda \in \mathbb {R}$, we have $G^*(B,C,\mathbf {V})=\infty$ unless $B=0$. Furthermore, it follows from the definition of $\mathcal {D}$ that $G^*(0,C,\mathbf {W})=\infty$ unless $C(t)\ge 0$ for a.e. $t\in [0,1]$.

For $B=0$ we have

$$\begin{aligned} G^*(0,C,\mathbf {W})&=\sup _{(A,\mathbf {V})\in \mathcal {D}}\left\{ \int _0^1 \tau ((\dot{A}(t)+\varepsilon \mathscr {L}A(t))C(t))\,dt\right. \\&\quad +\left. \int _0^1\langle \mathbf {V}(t),\mathbf {W}(t) \rangle _{\mathfrak {H}_{\mathcal {A},\mathcal {J}}}\,dt\right\} \\&\le \sup _{(A,\mathbf {V})\in \mathcal {D}}\bigg \lbrace -\int _0^1 \frac{1}{2}\Vert \mathbf {V}(t)\Vert _{C(t)}^2\,dt\\&\quad +\int _0^1\langle [C(t)]_{\varvec{\Lambda }}^{1/2} \mathbf {V}(t),[C(t)]_{\varvec{\Lambda }}^{-1/2}\mathbf {W}(t)\rangle _{\mathfrak {H}_{\mathcal {A},\mathcal {J}}}\,dt\bigg \rbrace \\&\le \frac{1}{2}\int _0^1\langle [C(t)]_{\varvec{\Lambda }}^{-1} \mathbf {W}(t),\mathbf {W}(t)\rangle _{\mathfrak {H}_{\mathcal {A},\mathcal {J}}}\,dt. \end{aligned}$$

We will show next that the inequalities are in fact equalities. Let $C^\delta =C+\delta$ and $\mathbf {V}^{\delta }(t)=[C(t)^{\delta }]^{-1}\mathbf {W}(t)$. Moreover, let $f_j=f_{\Lambda _j}$ with the notation from Lemma 4. Since

$$\begin{aligned} \mathcal {H}^{(h)}_\mathcal {A}\rightarrow \mathbb {R},\quad B\mapsto \sum _{j\in \mathcal {J}}\langle (df_{j}(C^{\delta }(t))B)\mathbf {V}^{\delta }_j(t),\mathbf {V}^{\delta }_j(t)\rangle _{\mathfrak {H}_\mathcal {A}} \end{aligned}$$

is a bounded linear map that depends continuously on *t*, there exists a unique continuous map $X^{\delta }:[0,1]\rightarrow \mathcal {H}^{(h)}_\mathcal {A}$ such that

$$\begin{aligned} \tau (B X^{\delta }(t))=\sum _{j\in \mathcal {J}}\langle (df_{j}(C^{\delta }(t))B)\mathbf {V}^{\delta }_j(t),\mathbf {V}^{\delta }_j(t)\rangle _{\mathfrak {H}_\mathcal {A}} \end{aligned}$$

for every $B\in \mathcal {H}^{(h)}_\mathcal {A}$ and $t\in [0,1]$.

Let

$$\begin{aligned} A^{\delta }:[0,1]\rightarrow \mathcal {A}_h,\quad A^{\delta } (t)=-\frac{1}{2}\int _0^t X^{\delta }(s)\,ds. \end{aligned}$$

We claim that $(A^{\delta },\mathbf {V}^{\delta })\in \mathcal {D}$. Indeed,

$$\begin{aligned} \tau (\dot{A}^{\delta }(t)\rho )&=-\frac{1}{2}\sum _{j\in \mathcal {J}} \langle (df_{j}(C^{\delta }(t))\rho )\mathbf {V}^{\delta }_j(t), \mathbf {V}^{\delta }_j(t)\rangle _{\mathfrak {H}_\mathcal {A}}\\&\le -\frac{1}{2}\sum _{j\in \mathcal {J}}\langle [\rho ]_{\Lambda _j} \mathbf {V}^{\delta }_j(t),\mathbf {V}^{\delta }_j(t)\rangle _{\mathfrak {H}_\mathcal {A}}\\&=-\frac{1}{2}\Vert \mathbf {V}^{\delta }(t)\Vert _{\rho }^2, \end{aligned}$$

where the inequality follows from Lemma 4. Note that we have equality for $\rho =C^{\delta }(t)$.

In particular, for $\rho =C(t)$ we obtain

$$\begin{aligned} \tau (\dot{A}^{\delta }(t)C(t))+\langle \mathbf {V}^{\delta }(t),\mathbf {W}(t)\rangle _{\mathfrak {H}_{\mathcal {A},\mathcal {J}}}\le \frac{1}{2}\langle [C(t)]_{\varvec{\Lambda }}^{-1}\mathbf {W}(t),\mathbf {W}(t)\rangle _{\mathfrak {H}_{\mathcal {A},\mathcal {J}}}. \end{aligned}$$

On the other hand,

$$\begin{aligned} \tau (\dot{A}^{\delta }(t)C(t))&=-\frac{1}{2}\sum _{j\in \mathcal {J}} \langle (d f_j(C^\delta (t))(C^\delta (t)-\delta ))\mathbf {V}_j^\delta (t), \mathbf {V}_j^\delta (t)\rangle _{\mathfrak {H}_A}\\&\ge -\frac{1}{2}\langle [C^\delta (t)]_{\varvec{\Lambda }}\mathbf {V}^\delta (t), \mathbf {V}^\delta (t)\rangle _{\mathfrak {H}_{\mathcal {A},\mathcal {J}}}+\frac{1}{2}\langle [\delta ]_{\varvec{ \Lambda }}\mathbf {V}^\delta (t),\mathbf {V}^{\delta }(t)\rangle _{\mathfrak {H}_{\mathcal {A},\mathcal {J}}}\\&\ge -\frac{1}{2} \langle \mathbf {V}^\delta (t),\mathbf {W}(t)\rangle _{\mathfrak {H}_{\mathcal {A},\mathcal {J}}}, \end{aligned}$$

where we again used Lemma 4 for the first inequality.

Put together, we have

$$\begin{aligned} \frac{1}{2}\langle [C^\delta (t)]_{\varvec{\Lambda }}^{-1}\mathbf {W}(t), \mathbf {W}(t)\rangle _{\mathfrak {H}_{\mathcal {A},\mathcal {J}}}&\le \tau (\dot{A}^{\delta }(t)C(t))+ \langle \mathbf {V}^{\delta }(t),\mathbf {W}(t)\rangle _{\mathfrak {H}_{\mathcal {A},\mathcal {J}}}\\&\le \frac{1}{2}\langle [C(t)]_{\varvec{\Lambda }}^{-1}\mathbf {W}(t),\mathbf {W}(t)\rangle _{\mathfrak {H}_{\mathcal {A},\mathcal {J}}}, \end{aligned}$$

and

$$\begin{aligned}&\lim _{\delta \searrow 0}\int _0^1 (\tau (\dot{A}^\delta (t)C(t))+\langle \mathbf {V}^\delta (t),\mathbf {W}(t)\rangle _{\mathfrak {H}_{\mathcal {A},\mathcal {J}}})\,dt\\&\quad =\frac{1}{2}\int _0^1 \langle [C(t)]_{\varvec{\Lambda }}^{-1}\mathbf {W}(t),\mathbf {W}(t)\rangle _{\mathfrak {H}_{\mathcal {A},\mathcal {J}}}\,dt \end{aligned}$$

follows from the monotone convergence theorem.

Hence

$$\begin{aligned} G^*(0,C,\mathbf {W})=\frac{1}{2}\int _0^1 \langle [C(t)]_{\varvec{\Lambda }}^{-1}\mathbf {W}(t),\mathbf {W}(t)\rangle _{\mathfrak {H}_{\mathcal {A},\mathcal {J}}}\,dt \end{aligned}$$

if $C(t)\ge 0$ for a.e. $t\in [0,1]$. Together with the formula for $F^*$, we obtainwhere the last equality follows from Proposition 1.

An application of the Rockafellar–Fenchel theorem yields the desired conclusion.
